# Serpentine Supravenous Hyperpigmentation in an HIV Patient Receiving R-CHOP for DLBCL

**Published:** 2018-01-01

**Authors:** Guido Lancman, Amir Steinberg, Janice Gabrilove

**Affiliations:** 1 Department of Medicine, Icahn School of Medicine at Mount Sinai, New York, NY, USA; 2 Tisch Cancer Institute, Mount Sinai Hospital, New York, NY, USA

**Keywords:** Serpentine supravenous hyperpigmentation, SSH, Chemotherapy, R- CHOP, DLBCL

## Abstract

Serpentine supravenous hyperpigmentation (SSH) is a rare vasculo-cutaneous entity that has been associated with peripheral infusion of chemotherapy agents, in particular 5-FU^1^^-^^3^, but also seen with docetaxel^4^^,^^5^, fotemustine^6^, and vinorelbine^7^. It consists of a marked hyperpigmentation along the superficial network of veins proximal to the chemotherapy infusion site and was originally described in a 1976 case report in association with 5-FU^1^. Here, for the first time, we report SSH in association with R-CHOP chemotherapy.

## Case presentation 

**Figure 1:**
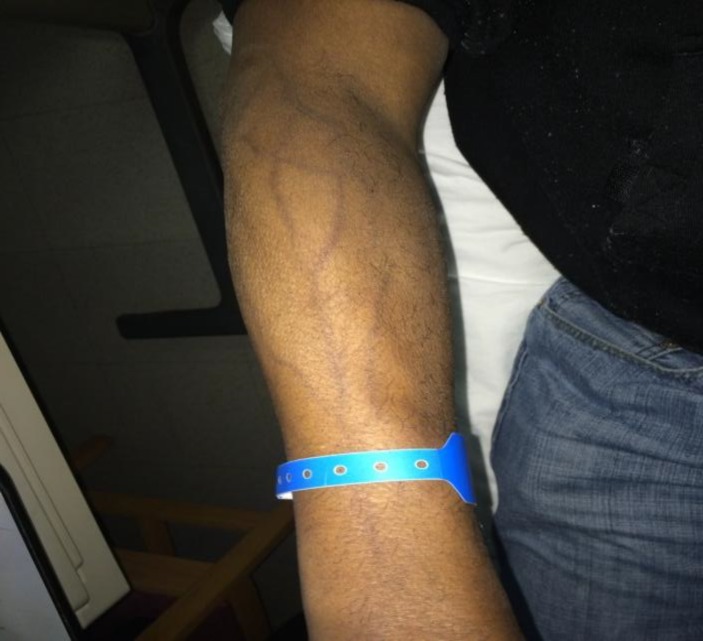
SSH in right arm following peripheral infusion of R-CHOP

A 50-year-old male with HIV, ESRD on PD, HTN, and recently diagnosed DLBCL presented to the Emergency Department with RUE pain and swelling ten days after receiving his first cycle of R-CHOP (cyclophosphamide dose reduced for PD) through a peripheral IV in the right hand. The patient reported that the swelling began in his right hand six days after the chemotherapy infusion, and was associated with tender, itchy, and notably darkened forearm veins. He also described shooting pains coming from the darkened veins. He denied any other rash or erythema, and endorsed chills but no fever. Physical examination was remarkable for mildly tender, deeply hyperpigmented veins originating at the site of his chemotherapy infusion site, and edema of the dorsum of the right hand without warmth or erythema (Figure 1). Labs were significant only for neutropenia (ANC 900). An upper extremity ultrasound was performed to rule out a DVT associated with this possible thrombophlebitis which was negative.

**Figure 2:**
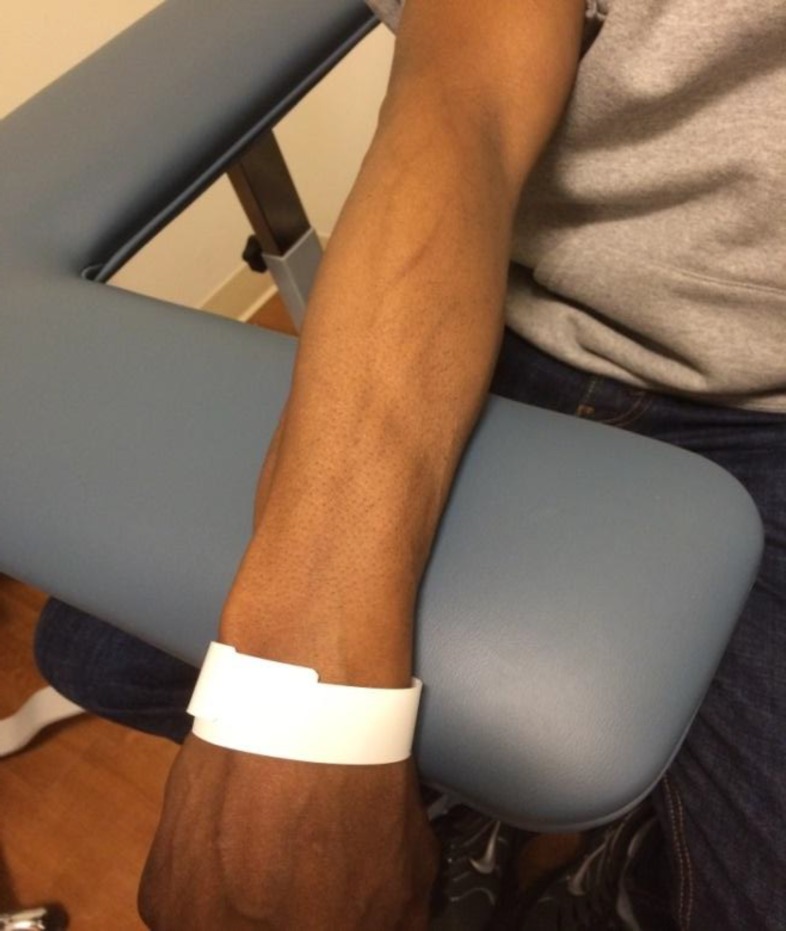
Near-resolution four months later

**Figure 3:**
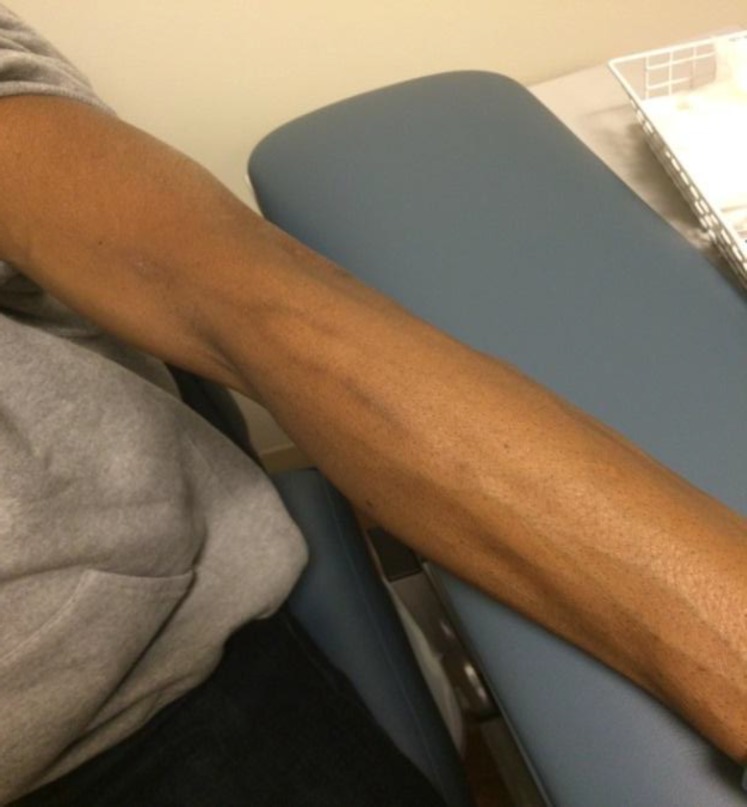
The second cycle of R-CHOP was peripherally

The patient was diagnosed with serpentine supravenous hyperpigmentation (SSH) and discharged home with supportive care on hospital day 2. Upon follow-up in the office four months later, the original SSH in his right arm had improved significantly (Figure 2), but he developed new SSH in his left arm where he was now getting his chemotherapy infusions (Figure 3). 

In this instance, however, he did not feel any pain or discomfort from the darkened veins. 

## Discussion

 SSH is a rare and poorly understood entity usually presenting in the setting of recent chemotherapy infusion. Dermatopathology has shown that there is an increase in melanin production without destruction of basal cells or inflammatory infiltrate^6^ . This is distinguished from persistent supravenous erythematous eruption (PSEE), which presents clinically with an erythematous rash and histologically with vacuolar alteration of basal cells and perivascular inflammatory infiltrate^        8 ^. PSEE lesions can eventually become hyperpigmented and take on the appearance of SSH. The reasons have not yet been elucidated, but it is thought to be a reaction to the cytotoxic drugs as opposed to direct extravasation. In addition, there have been reports of PSEE/SSH in patients treated for leprosy ^9^^,^^10^ , autoimmune hemolytic anemia, and HIV^   12^ although in these cases the distribution is usually bilateral and unrelated to any peripheral infusions. 

Based on the published case reports, SSH can appear anywhere between a few hours and several weeks after infusion of chemotherapy. Management consists of recognition and symptomatic treatment; a Doppler ultrasound can be performed if there is suspicion for thrombophlebitis. The rash usually resolves within 1-3 months. In this case, resolution of the original SSH was noted in the patient’s right arm, however upon switching chemotherapy infusions to the other arm he developed new SSH at that site. This suggests that certain patients may be intrinsically more prone to developing this reaction, regardless of the infusion site.

## CONCLUSION

 Serpentine supravenous hyperpigmentation is a rare vasculo-cutaneous entity most commonly associated with peripheral chemotherapy infusions but also seen in other conditions such as leprosy, autoimmune hemolytic anemia, and HIV. Here we described the first case of SSH after peripheral infusion of R-CHOP chemotherapy. Prompt recognition is important to ensure that a superficial thrombophlebitis is ruled out and to avoid further unnecessary testing. Treatment is supportive as SSH usually resolves within 1-3 months. 
